# An Immune Atlas of T Cells in Transplant Rejection: Pathways and Therapeutic Opportunities

**DOI:** 10.1097/TP.0000000000004572

**Published:** 2023-10-21

**Authors:** Sarah Short, Guido Lewik, Fadi Issa

**Affiliations:** 1 Nuffield Department of Surgical Sciences, University of Oxford, Oxford, Oxfordshire, United Kingdom.

## Abstract

Short-term outcomes in allotransplantation are excellent due to technical and pharmacological advances; however, improvement in long-term outcomes has been limited. Recurrent episodes of acute cellular rejection, a primarily T cell–mediated response to transplanted tissue, have been implicated in the development of chronic allograft dysfunction and loss. Although it is well established that acute cellular rejection is primarily a CD4^+^ and CD8^+^ T cell mediated response, significant heterogeneity exists within these cell compartments. During immune responses, naïve CD4^+^ T cells are activated and subsequently differentiate into specific T helper subsets under the influence of the local cytokine milieu. These subsets have distinct phenotypic and functional characteristics, with reported differences in their contribution to rejection responses specifically. Of particular relevance are the regulatory subsets and their potential to promote tolerance of allografts. Unraveling the specific contributions of these cell subsets in the context of transplantation is complex, but may reveal new avenues of therapeutic intervention for the prevention of rejection.

## INTRODUCTION

Transplantation is a lifesaving intervention for end-stage organ disease, yet adaptive immune responses to the engrafted tissue are a significant limitation. Although the rate of acute allograft rejection has improved significantly because of technical refinements and modern immunosuppressive therapy, immune-mediated rejection remains a significant cause of early allograft loss and chronic graft dysfunction.

The alloresponse to a transplanted graft has recently been reviewed.^1^ Traditionally, acute rejection events are classified as either antibody-mediated rejection (AMR) or acute cellular rejection (ACR). ACR is composed of 2 primary phases, allorecognition of nonself-antigens by the host immune system and the ensuing effector response, which leads to graft injury, culminating in rejection. ACR is a primarily CD4^+^ and CD8^+^ T cell–mediated response to nonself major histocompatibility complex (MHC) molecules. The T cell compartment is profoundly heterogenous, as the diverse cytokine environments antigen-primed CD4^+^ T cells differentiate within influence the subsequent subset commitment. The function and relevance of these subsets within the context of transplantation are complex and, in some cases, poorly defined.

Clarifying the specific contributions of distinct T cell subsets may unveil effective therapeutic routes to further minimize ACR occurrence. This is of particular importance because although rates of acute graft loss within the first year are low, there is accumulating evidence to suggest that repeated episodes of subclinical ACR increase the risk of later allograft failure, particularly in renal allotransplantation.^2,3^ In this review, we provide an overview of the mechanisms by which T cells promote and control ACR, highlighting potential therapeutic targets.

## CD4^+^ T CELL SUBSETS

CD4^+^ T cells play a central role in ACR. Naïve CD4^+^ T cells are activated and subsequently differentiate into specific T helper (Th) subsets under the influence of the local cytokine milieu generated by innate and stromal cells during immune responses. The strength of T cell receptor (TCR) stimulation has also been shown to help direct the differentiation of naïve T cells.^4^ It is important to note that although previously regarded as a permanent commitment once differentiated, it is now widely accepted some cells may retain a level of plasticity, facilitating conversion to other lineages under certain conditions.^5^

**FIGURE 1.**
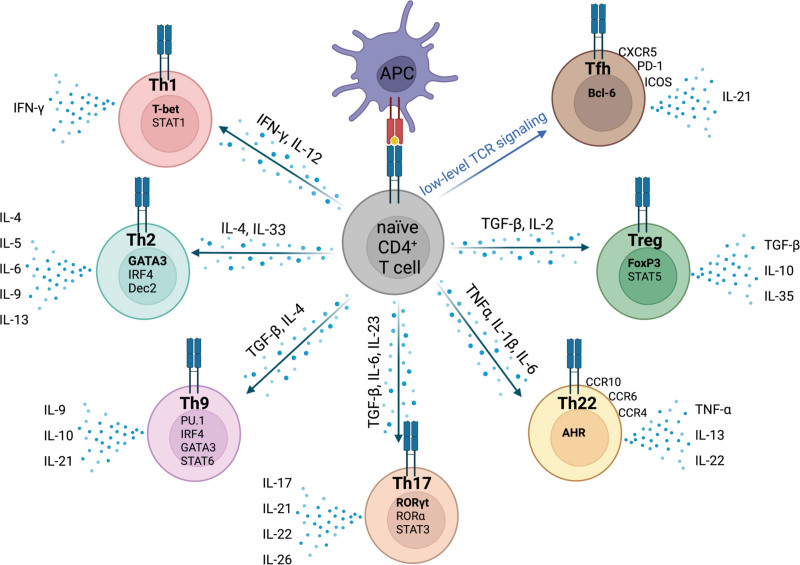
Development of CD4^+^ T-cell subsets and their phenotypic markers. Naive CD4^+^ T cells differentiate into phenotypically and functionally distinct subsets upon MHC-II-dependent activation via antigen-presenting cells. TCR signaling strength determines development of Teff or Tfh cells. Subtype lineage fate is driven by the cytokine environment (not shown for Tfh subsets). Each subset is displayed with characteristic markers. Created with BioRender.com. IFN-γ, interferon gamma; IL, interleukin; IRF4, interferon-regulatory factor 4; MHC, major histocompatibility complex; ROR, RAR-related orphan nuclear receptor; TCR, T-cell receptor; Teff, T effector; Tfh, T follicular helper; TGF-β, transforming growth factor beta; Th, T helper cell; TNF-α, tumor necrosis factor alpha; Treg, regulatory T cell.

After the initial characterisation of Th1 and Th2 subsets in the seminal paper by Mosmann et al^6^, the initial paradigm has expanded to encompass Th17, Th9, Th22, T follicular helper (Tfh), T follicular regulatory (Tfr), and regulatory T cell (Treg) lineages. The majority of these cells have distinct transcription factor signatures, cytokine production profiles, and effector functions, which enable their delineation (Figure 1). Each subset has been implicated to varying degrees in the mediation or abrogation of alloimmune rejection processes. However, despite several decades of active investigation, their specific roles in transplantation are yet to be completely understood.

### CD4^+^ Th1 Subtype

As the first group of differentiated CD4^+^ T cells to be characterized, Th1 cells have well-established roles in the clearance of intracellular pathogens and pathogenesis of autoimmune disease. Activation of naive T cells in the presence of interleukin (IL)-12 and interferon-gamma (IFN-γ) results in activation of signal transducer and activation of transcription (STAT) 1 and the expression of the Th1 specific transcription factor T-bet, which directs Th1 lineage commitment and regulates the expression of IFN-γ by Th1 cells.^7^ Potent secretion of interferon IFN-γ is a hallmark of the subset.

Th1 cells were traditionally regarded as largely responsible for autoimmune and rejection responses. However, observation of persistent experimental autoimmune encephalitis pathology in IFN-γ–knockout mice, which was previously considered a Th1-mediated pathology, challenged this assumption.^8^ It was subsequently observed that IL-23 promoted a distinct IL-17–producing CD4^+^ activation state,^9^ now recognized as the Th17 profile. Consequently, there has been a focus on clarifying the relative roles of Th1 and Th17 in inflammation and rejection in recent y.

Th1 cells promote alloimmune responses in the graft through secretion of proinflammatory IL-2, IL-12, IFN-γ, and tumor necrosis factor-alpha (TNF-α), which promote leukocyte recruitment and cytotoxic T lymphocyte (CTL) priming.^10^ Physiologically, IFN-γ has a range of actions, including stimulation of macrophage activity, promotion of antigen processing and presentation, orchestration of leukocyte attraction, and enhancement of natural killer (NK) cell activity.^11^ In a transplant context, IFN-γ promotes T-cell infiltration of the allograft through monokine induction, promoting acute rejection mechanisms.^12^ A positive correlation between intragraft IFN-γ and acute rejection has been reported in patient populations, although IL-17 expression has also been reported, indicating the possibility of a shared role.^13^ More specifically, an association between a pretransplant Th1 cytokine pattern and early acute rejection events has been described^14^ and supported by a meta-analysis of pretransplant IFN-γ Enzyme-linked immunosorbent spot results.^15^ Overall, despite ongoing efforts to unravel the overlapping roles of Th1 and Th17 subsets, the Th1 compartment appears to promote acute rejection mechanisms.

### CD4^+^ Th2 Subtype

In the context of transplantation, Th2 cells have traditionally been attributed with graft-protective properties in contrast to proinflammatory Th1 cells.^16^ The Th2 subset is characterized by a cytokine profile comprising IL-4, IL-5, IL-6, IL-9, and IL-13.^17^ Development and lineage commitment depends on the transcription factor GATA binding protein 3,^18^ interferon-regulatory factor 4 (IRF4), and Dec2.^19^

Representing the prototypic cytokine of the Th2 subset, IL-4 has key roles in regulation of inflammation, induction of macrophage activity, and the development of effector T-cell responses. Although antibody production was initially attributed to broadly classified Th2 cells, further delineation of CD4^+^ subsets and recent in vivo work have revealed that Tfh-derived IL-4 drives germinal center (GC) responses and, specifically, IgE and IgG1 antibody production.^20^ Mechanistically, both IL-4 and IL-13, functionally similar cytokines, have been found to antagonize proinflammatory TNF-α and IFN-γ signaling and promote antioxidant and antiapoptotic functions.^21,22^ IL-13 may also impair T cell infiltration by reducing expression of migratory E-Selectin and Monocyte chemoattractant protein-1.^22^ In various preclinical transplantation models, IL-4 treatment or secretion has been shown to promote allograft survival, which was attributed to promotion of Th2 responses and reduction in Th1 frequency,^23^ whilst also expanding the Treg compartment.^24^ Additionally, promotion of Th2 subset predominance, whether intentional or as an off-target effect of an intervention, has been shown to ameliorate rejection in several mouse models.^25-27^ An association between improved graft health and lowered rejection grades and a Th2-favoring shift in the Th1/Th2 balance has been demonstrated, emphasizing the potential importance of this subset balance.^28^ Supporting the importance of this balance, increased Th1/Th2 ratios have been observed in rejecting mouse corneal allografts,^29^ whereas a shift toward Th2 predominance via impaired TGF-β (transforming growth factor beta) signaling could ameliorate allograft rejection.^25^

Several modulators of Th2 activity in transplantation have been described. Clinically, disruption of the Th2 transcription factor NLR family pyrin domain containing 3 has been shown to abrogate Th2 responses and was associated with reduced cardiac allograft survival.^30^ In contrast, a comparative study of 34 renal transplant recipients found that decreased infiltrating Th1/Th2 ratios correlated with plasma cell-rich rejection, which is consistent with observed promotion of plasma cell differentiation from B cells via Th2 cytokines.^6,31^ The costimulatory molecule T cell immunoglobulin domain and mucin-domain-4 (TIM4) also regulated Th2 differentiation in some preclinical models.^32^ Blocking TIM4 has extended allograft survival through reduced Th2 differentiation in a mouse hepatic transplant model.^33^ Additionally, adoptive transfer of Th2 cells has induced rejection in several murine transplant models, including skin and islet allografts,^34,35^ supporting the role of Th2 cells in rejection and their potential as a therapeutic target. Therapeutic targeting of Th2 cells with largely graft-protective results appears to contradict literature suggesting a tolerogenic role. However, the efficacy of these interventions may reflect a correction of an underlying pathological Th1/Th2 imbalance.

### CD4^+^ Th9 Subtype

Th9 cells are a more recently identified subset implicated in a range of inflammatory diseases, although their role in the context of transplantation is unclear. The Th9 compartment develops from naïve CD4^+^ cells in the presence of TGF-β and IL-4 in vitro,^36^ and requires the transcription factors STAT6,^36^ PU.1,^37^ IRF4, and GATA binding protein 3.^38^ The population is characterized by potent production of IL-9, in addition to IL-10 and IL-21.

A consensus on the role of Th9 cells and IL-9 in transplantation has not been reached, with conflicting reports of both protolerogenic and detrimental roles within the literature. In addition to the general paucity of data, most of the studies of Th9 cells in transplantation are preclinical models, which are often difficult to accurately translate to clinical contexts. Lu et al^39^ have reported a protective role of IL-9, demonstrating higher levels of IL-9 in tolerant allografts, which promoted Treg suppressive activity and consequent allograft tolerance. Neutralization of IL-9 significantly accelerated skin allograft rejection in tolerant mice, supporting this conclusion.^39^ A similar IL-9-mediated promotion of Treg suppression has also been reported in a mouse skin allograft model.^40^ Although the role of Th9 cells was not specifically addressed, the results indicate protolerogenic potential.

Elyaman et al^41^ have reported enhanced suppressive capacity of natural Treg in vitro and in vivo via IL-9, supported by findings from Eller et al^42^ who postulated that this effect occurs through IL-9–mediated mast cell recruitment. Paradoxically, IL-9 also directs differentiation of naïve CD4^+^ T cells into a proinflammatory Th17 phenotype in the presence of TGF-β,^41^ indicating rejection potentiating capacity. Transgenic overexpression of IL-9 in a mouse cardiac transplantation model accelerated rejection, whereas IL-9 deficiency did not affect survival compared with wild-type mice.^43^ CD96 expression has been proposed as a potential indicator of Th9 inflammatory capability. Two distinct populations of Th9 cells have been identified, CD96^high^ and CD96^low^, with adoptive transfer of the latter inducing significant colitis and skin allograft rejection in mouse studies.^44^

These results have generated interest in the possibility of a correlation between serum IL-9 levels and allograft tolerance. Although higher serum IL-9 levels among human transplant recipients with lower immunosuppressive load have been reported,^45^ it was later established that there is no difference in levels between rejecting and stable patients.^46^

### CD4^+^ Th17 Subtype

First established as a separate lineage to Th1 and Th2 cells in 2005,^47,48^ Th17 cells have since been recognized for their significant roles in the induction of autoimmunity and tissue inflammation. Th17 cells differentiate from naïve CD4^+^ T cells in the context of inflammatory cytokine milieu after TCR stimulation. Differentiation is regulated by the lineage-specific transcription factor RAR-related orphan nuclear receptor-γt and RAR-related orphan nuclear receptor-α,^49,50^ and promoted by TGF-β, IL-6, and IL-21 via STAT3 signaling in vitro.^51^ IL-23 is required for the expansion and stabilization of the population.^52^ Once induced, Th17 cells are characterized by potent secretion of IL-17, IL-21, IL-22, and IL-26 cytokines.

The IL-17 family encompasses 6 structurally related cytokines (IL-17A-F), with IL-17A being the most well defined. IL-17A signals through the IL-17RA receptor on target cells, a shared receptor for the different IL-17 isoforms, promoting inflammatory responses via activation of pathways including mitogen-activated protein kinase and nuclear factor κ B.^53^ Although regulated IL-17 secretion has physiological roles, including tissue repair, prolonged secretion can promote pathological inflammation through activation of proinflammatory signaling pathways and robust neutrophil induction.^54^ IL-17 is the pathognomonic cytokine of Th17, however it is also secreted by other cell types, including NK, γδ T, and other CD4^+^ subsets, making it difficult to definitively attribute IL-17 action to Th17 function.

There is evidence for a role of IL-17 in acute allograft rejection as early as 1999.^55^ Multiple studies have demonstrated prolonged cardiac allograft survival and inhibition of T cell responses to alloantigen in mouse models of IL-17 antagonism.^55,56^ Allospecific Th17 cells have also been implicated in the pathogenesis of acute lung allograft rejection in both mouse^57^ and small cohort clinical studies.^58^ Overexpression of IL-17A in mice undergoing acute lung rejection, with attenuation of rejection severity with IL-17 deficiency, offers further support for a central role for Th17 cells.^59,60^ Clinically, a temporary upregulation of IL-17 has been demonstrated in human lung transplant recipients undergoing acute rejection,^61^ and a positive correlation between Th17 frequency and rejection activity in hepatic transplant patients has been reported.^62^ These reports indicate that the extensive preclinical observations supporting the role of IL-17 in rejection mechanisms may hold true in humans.

Several studies have explored the relationship between Treg and Th17 cells, emphasizing the importance of the Treg/Th17 ratio in prevention of allograft rejection.^63^ Zhou et al^64^ demonstrated attenuation of acute rejection and prolonged survival of allografts in a mouse lung transplantation model by shifting the balance of this ratio through adoptive transfer of induced Treg. The authors attributed this effect to the downregulation of Th17 and IL-17^+^ γδ T cells, reduced IL-17 production, and enhanced protolerogenic IL-10 secretion.^64^ Similarly, a single-center study of liver transplant recipients identified a significant elevation of Th17/Treg ratio in patients undergoing acute allograft rejection compared with stable recipients and pretransplant levels.^65^ Notably, there was a significant correlation between serum IL-17 levels and Th17 cell frequency.^65^

### CD4^+^ Th22 Subtype

First described by Eyerich et al^66^, the differentiation of CD4^+^ T cells into Th22 cells is directed by IL-6, IL-1β, TNF-α, and aryl hydrocarbon receptor (AHR) ligation.^66,67^ Th22 cells can be induced by plasmacytoid dendritic cells^67^ and Langerhans cells.^68^ The subset is typically defined by AHR expression; phenotypic markers CCR4^+^, CCR6^+^, and CCR10^+^; and production of IL-22.^69^ Notably, although Th17 cells also secrete IL-22, Th22 cells do not exhibit notable levels of IL-17 production.^66,67^ Both proinflammatory and anti-inflammatory functions have been demonstrated by the Th22 helper T cell subset in allotransplantation.

Predominately investigated in skin disorders, the role of Th22 cells currently remains largely ill-defined in the context of transplantation.^70,71^ Several preclinical studies have connected rejection to IL-22 and Th22 cells specifically, however. In a nerve xenotransplantation model, an increase in the ratio of Th1, Th17, and Th22 cells to Treg in the spleen and elevated IFN-γ, IL-17, and IL-22 in the serum was found during acute rejection.^72^ Neutralization of these cytokines resulted in significantly prolonged graft survival.^72^ Allogeneic stem cell transplantation in mice has also been shown to evoke a proinflammatory Th22 cell differentiation that was promoted by IL-17, yet arose independently from the Th17 lineage,^73^ which is consistent with in vitro findings.^74^

The role of T cell subsets, including Th22 cells, in graft-versus-host disease (GvHD) has recently been reviewed.^75^ In brief, IL-22 has been associated with increased severity of GvHD, possibly through induction of STAT3, which may promote graft lymphocyte infiltration,^76^ expansion of effector T cell populations, and reduction of Treg frequency.^77^ Supporting this disease-potentiating role, IL-22-deficient donor T cells have been shown to reduce the severity of GvHD in mice, accompanied by increased Treg numbers.^76^ Consequently, it has been proposed that the IL-22-STAT3 pathway may contribute to GvHD tissue injury and could serve as a therapeutic target.

Although the limited data available are suggestive of Th22 participation toward allograft rejection, conflicting studies exist, attributing regulatory function to IL-22 in experimental GvHD mouse models.^78^ In clinical patients with steroid-refractory GvHD, complete responders to extracorporeal photopheresis demonstrated increased Th22 cell frequencies, whereas Th22 cells were decreased in nonresponders,^79^ indicating a potential regulatory role.

## Tfh CELLS

Tfh cells are a distinct CD4^+^ subset with essential roles in supporting effective B lymphocyte humoral responses, particularly within GCs. Tfh cells are characterized by expression of CXCR5, which facilitates B cell follicle homing, PD-1, and ICOS.^80^ The transcription factor Bcl-6 is essential for Tfh differentiation,^81^ whereas IL-6, IL-21, and IL-12 influence differentiation through STAT3, STAT1, and STAT4, respectively.^82^ IL-21 is the archetypal cytokine secreted by the subset. Recent studies have substantially informed our understanding of Tfh cells, which develop on low-level TCR signaling into distinct populations depending on cytokine environment, thereby mirroring CD4^+^ effector subsets. Tfh equivalents of each Teff (T effector) subtype have therefore been described to reflect a different activation state.^83^

Tfh cells are postulated to play a role in transplant alloimmunity and allosensitization mechanisms and are best characterized in the setting of renal transplantation. In kidney transplant recipients, formation of donor-specific alloantibodies is a major cause of graft loss. Tfh cells provide help to alloantigen-specific B cells that markedly shape antibody responses. Monitoring of circulating Tfh (cTfh) cell subset distribution and phenotype as a surrogate of Tfh activation, and therefore antibody responses, has shown promise after vaccination and in patients with autoimmune disease.^84^ Consequently, several studies have focused on a potential correlation between elevated numbers of cTfh and de novo donor-specific antibody (DSA) formation in transplant recipients.^85,86^ Although, notably, other authors have observed no difference in cTfh numbers between DSA^+^ and DSA^–^ patients.^87^

The formation of ectopic lymphoid aggregates with varying organization, termed tertiary lymphoid organs (TLOs), has been reported within allografts in recent years. These structures typically demonstrate segregated T and B zones and dendritic cell networks reminiscent of GCs. Whether these TLOs are protective, destructive, or unrelated to allograft survival remains debated within the literature. Although some studies suggest that TLOs accelerate graft loss, others argue the presence of regulatory cells within these structures suggests a tolerogenic function.^88,89^ Immunohistochemical analyses of renal allografts have demonstrated colocalization of Bcl-6, IL-21, and Ki67 predominately within these T and B cell aggregates, indicating a potential role for activated Tfh cells in TLO mechanisms.^90^ Thaunat et al^91^ have also reported a significant Tfh signature within these TLOs in chronically rejecting murine cardiac allografts, suggesting a key role of Tfh cells in the coordination of local humoral responses. It remains too early to conclude whether Tfh cells within these TLOs are contributing to tolerogenic or rejection mechanisms, as evidence exists for both.

Choice of induction therapy has been shown to distinctly alter the Tfh phenotype after renal transplantation,^87^ with certain agents skewing polarization to a Th1-like phenotype.^92^ Consistent with these findings, a concordance between cTfh phenotype and DSA formation and GC alloreactivity has been noted in preclinical models, proposing their potential as biomarkers of humoral reactivity and DSA formation after transplantation.^86^ Several studies have also suggested dysregulation of Foxp3^+^ Tfr, which regulate B and Tfh activity, may play a role in renal allograft dysfunction.^87,92^

## CD8^+^ CYTOTOXIC T CELLS

CD8^+^ T cells, or CTLs, represent the key effector subset in adaptive immunity and play a central role in allograft destruction after direct activation via MHC class I alloantigen presentation. CTLs may be delineated into naïve, central memory, Terminally differentiated effector memory subsets based on their expression of CD45RA and the lymphoid homing receptor CCR7.^93^ Graft-infiltrating CTLs exert their cytotoxic action via various mechanisms, including secretion of proinflammatory TNF-α and IFN-γ, perforin and granzyme-induced apoptosis of the target cell, Fas/FasL interactions, and direct invariant NK cell activation.^94^ Migration of alloreactive CTLs depends on cognate antigen presentation rather than Gα_i_-coupled chemokine receptor signaling, the main mechanisms for antigen-nonspecific bystander CD8^+^ T cells, as demonstrated in mouse models of heart and kidney transplantation.^85,86^ Recent in vivo studies report that allogeneic CD8^+^ T cell effector differentiation hinges on transcription factors BATF and IRF4, with respective deficiencies promoting skin graft survival in adoptive cell transfer experiments.^95-97^ These markers may thus be of interest for therapeutic targeting in future studies.

In renal transplant recipients, CTLs are critical effectors of allograft injury. The extent of CTL infiltration appears to correlate with allograft survival^98,99^ and granzyme B levels are positively associated with the extent of graft damage.^100,101^ Notably, although perforins are not required for CTL-mediated rejection, IFN-γ is essential.^102^ Transplant studies incorporating immunomonitoring may provide insights into the CD8^+^ subsets involved in rejection response. Risk of acute rejection has been correlated with increased frequency of effector memory CTLs^103^ and Terminally differentiated effector memory populations.^104^ Renal transplant recipients exhibiting a high pretransplant frequency of CD8^+^ T cells highly expressing the CD45RC isoform, which may identify an alloreactive subset, had a significantly increased risk of rejection.^105^ Antibody-mediated depletion of this subset with anti-CD45RC was capable of inducing donor-specific transplant tolerance in a rodent cardiac allotransplant model, with preservation of general immunity.^106^ This example demonstrates the value of identifying specific cell subsets for therapeutic targeting with the ultimate goal of reducing off-target immunosuppression.

CTLs demonstrate important interactions with several other cell types in transplantation. Despite direct allo-MHC-I activation being the main mechanism to evoke graft damage, animal models have demonstrated CD4^+^ T cell–dependent cytotoxic CD8^+^ responses early after transplant.^98,99^ It has been proposed that activation of directly alloreactive CTLs may occur by nonhematopoietic cells,^107^ which may be the result of semidirect MHC acquisition by recipient Antigen-presenting cells (APCs).^108^

Established hypotheses propose the ability of CTLs to evoke a Th1 cytokine intragraft environment in vivo while inhibiting Th2 cytokine production.^109^ Reciprocal activation of CTLs and Th1 cells via IFN-γ and IL-2, respectively, was also found to promote inflammatory responses.^108^ In particular, the CTL/Treg balance appears to be crucial, as evidenced by borderline cases of rejection in renal transplant patients where Treg seem to prevent acute rejection by counterbalancing CTL infiltration.^110^ Data on the influence of immunosuppressive regimens remain limited in this context, and no significant difference in intragraft CTL markers was found when comparing costimulatory blockade using belatacept with calcineurin inhibitor treatment at 1 year post kidney transplant.^111^

Despite their overwhelmingly deleterious effects, recent single-cell RNA sequencing results demonstrated CTL conversion to a more regulatory phenotype within 3 wk in accepted mouse kidney allografts, attesting to plasticity in certain conditions.^112^ Further investigations are warranted to investigate these findings.

## REGULATORS

Treg subsets are a heterogenous cohort encompassing CD4^+^Foxp3^+^ cells, Tfr cells, and CD8^+^ regulatory cells, characterized by their critical role in the promotion of peripheral tolerance. The CD4^+^Foxp3^+^ subset can be further delineated into several distinct compartments, which has been discussed in a recent review^113^ and goes beyond the focus of this article.

### CD4^+^ Treg

Recognized as thymically derived CD4^+^CD25^+^ T cells with tolerogenic capacity, understanding of the compartment expanded rapidly with the discovery of Foxp3 as the master transcription factor in 2003.^114^ Treg are broadly classified as thymically derived “natural” Treg or induced/peripherally derived Treg, which develop from naive CD4^+^ T cells in the presence of TGF-β and repeated antigenic stimulation. Treg mediate peripheral tolerance indirectly through secretion of anti-inflammatory TGF-β, IL-10, and IL-35 and directly via granzyme-mediated cytotoxicity, inhibition of APC activation, CD39-mediated hydrolysis of extracellular ATP, and metabolic disruption via competitive consumption of IL-2.^115,116^ Treg constitutively express high levels of CD25, the IL-2 receptor α chain, which together with CD133 and CD122 forms a 3-subunit receptor configuration, conferring a profound increase in receptor affinity for IL-2. IL-2 is essential for the promotion of survival and expansion of both Teffs and Treg in vivo.^117^ In proinflammatory microenvironments, competitive consumption of IL-2 effectively deprives Teffs of the IL-2 required for cell survival and proliferation, inducing apoptosis.^118^

Both clinical and preclinical studies have demonstrated the ability of Treg to modulate graft rejection after solid organ transplantation, reduce severity of GvHD, and control pathogenic effector responses in various autoimmune contexts.^116,119-121^ Effective migration of Treg to the graft site has been shown to be critical in achieving effective suppression of local graft rejection processes preclinically.^122,123^ Although the large extent of research focuses on polyclonally expanded Treg, several preclinical humanized transplant models have reported a protective advantage of donor alloantigen-reactive Treg.^124,125^ Several human clinical trials have since used donor alloantigen-reactive Treg although it is unclear if they offer superior outcomes.^126^

There is an increasing body of evidence for the modulation of undesired immune responses by adoptive transfer of Treg, with the ultimate goal of inducing allograft tolerance. Several phase I/IIa clinical trials have been completed in recent y with encouraging results, demonstrating safety and fewer infectious complications, although similar rejection rates.^127^ A phase IIb trial is currently underway (ISRCTN11038572). An alternative approach is the induction of Treg through low-dose IL-2 treatment, which has shown promise in several preclinical models and offers a more cost-effective solution when compared with cellular therapies.^128^ Numerous clinical trials have been completed in the context of autoimmune disease however, fewer have been attempted in transplant recipients. A recently reported small cohort trial in liver transplant recipients found that low-dose IL-2 therapy selectively increased circulating Treg frequency yet failed to induce tolerance and was terminated early because of safety concerns.^129^ The authors attributed this failure to increased liver immunogenicity because of off-target effects.^129^ There is current interest in IL-2 muteins with the hope that they could offer efficacy with fewer off-target effects however, no such agents have been trialed in humans to date in the context of transplantation.

## FOLLICULAR REGULATORY CELLS

Tfr cells arise from thymically derived CD25^hi^ Foxp3^+^ Treg precursor cells in a Tfh-like differentiation mechanism, dependent on Bcl-6, SPA, ICOS, CD28, and B cells.^130^ They develop and maintain a CD4^+^Foxp3^+^Bcl-6^+^Blimp1^+^ phenotype, upregulate CXCR5 upon priming by Dendritic cells, and migrate to B cell follicles where they may suppress GC responses.^131,132^

It has been postulated that the balance of antigen-specific Tfr and Tfh cells could be crucial for the development of DSA and AMR. Interestingly, in mice, this balance shifts toward Tfr cells upon immunization with self-antigen and toward Tfh cells upon nonself-antigen presence, as found in the allotransplant setting.^133^ Recently, Tfr cells were reported to inhibit de novo DSA formation upon alloantigen challenge, yet only had subtle effects on the course of rejection in mouse renal allotransplants.^134^ Clinically, kidney transplant patients with AMR and chronic allograft dysfunction often exhibit decreased circulating Tfr frequencies, suggesting these cells may play an important role in allograft tolerance.^135,136^ An increased Tfr/Tfh ratio could thus be beneficial for allograft survival and future studies that delineate underlying mechanisms may aid in identifying interventions that favorably tilt the balance.

### CD8^+^ Treg

Historically, CD8^+^ Treg were the first described cell subset with suppressive capacity.^137^ However, the lack of specific markers to identify this distinct population of CD8^+^ cells limited more extensive research for many y. CD8^+^ Treg are thought to abrogate allograft rejection through inhibition of antigen-specific T lymphocyte proliferation and direct cell-to-cell mechanisms. In vitro, CD8^+^ Treg mediate the activity of cytotoxic lymphocytes by decreasing HLA Class I expression on target cells in an IL-10–dependent manner.^138^ This compartment also induces tolerogenic APCs via upregulation of inhibitory receptors immunoglobulin-like transcripts (ILT)3 and ILT4, and downregulation of costimulatory molecules such as CD80 and CD86.^139^ These tolerogenic APCs are rendered capable of inducing antigen unresponsiveness in CD4^+^ Th cells. Chang et al^139^ observed a strong association between the absence of acute rejection and the capacity of CD8^+^ Treg to induce upregulation of ILT3 and ILT4 on APCs in a small cohort of cardiac allograft recipients, indicating the importance of this immunosuppressive mechanism.

The suppressive capacity of CD8^+^ Treg is supported by several preclinical transplantation models. Prolongation of allogeneic pancreatic allograft and skin graft survival has been reported after adoptive transfer of induced allospecific CD8^+^ Treg^140^ and expanded CD8^+^ Treg, respectively.^141,142^ Similarly, CD8^+^ Treg induced via stimulation of CD3^+^ T cells with allogeneic plasmacytoid Dendritic cells are reportedly capable of inhibiting T cell alloresponses, including memory T-cell responses.^143^ These preclinical findings demonstrate the therapeutic potential of adoptive CD8^+^ Treg transfer, and although there is interest in human clinical trials, none have been completed to date.^144^

## UNCONVENTIONAL T CELLS

### Gamma–Delta T Cells

Gamma–delta (γδ) T cells comprise a heterogeneous compartment of unconventional lymphocytes, expressing heterodimeric TCRs composed of γ and δ chains. They are functionally diverse, exhibiting both innate and adaptive behaviors spanning direct cytotoxicity and immunomodulation.^145^ γδ T cells constitute 1% to 5% of circulating lymphocytes but can undergo rapid expansion in response to malignancy, inflammation, and infection.^146^ The plasticity of γδ T cells in the tissue microenvironment facilitates their functional diversity.

γδ T cells play a role in delivering rapid stress-surveillance responses triggered by threats to tissue integrity. There is limited evidence that the subset may play a role in abrogating tissue damage in ischemia–reperfusion injury (IRI). IRI is the primary inducer of innate immunity in the early posttransplant phase, enhancing acute allograft rejection mechanisms. A significant reduction in renal tubular necrosis was reported when kidney transplantation was performed in TCR γδ-deficient mice.^147^ More recently, selective deficiency of γδ T cells was shown to attenuate the inflammatory response to acute intestinal IRI in a mouse model. An associated reduction in hepatic biomarkers provides preliminary evidence that this deficiency may also ameliorate distant organ injury.^148^

Unsurprisingly, given the abundance of evidence for a key role of IL-17 in allograft rejection, infiltration of IL-17–producing γδ T cells has been associated with promotion of allograft rejection,^149,150^ and depletion associated with attenuation of rejection.^151^ In addition to prolonged allograft survival, depletion of γδ T cells has also been associated with enhanced accumulation of immunoregulatory Foxp3^+^ Treg.^151^

In humans, γδ T cells are broadly classified as Vδ2 positive or negative, based on the TCR δ chain. The Vδ2 cells are the predominate circulating subset, comprising >70% of γδ T cells in peripheral circulation. The primarily tissue-resident Vδ2^neg^ γδ T cells are present at higher frequency at epithelial and mucosal barriers.^145^ Vδ2^neg^ γδ T cells have key roles in immunosurveillance, producing potent inflammatory responses upon tissue dysregulation and viral infection. Vδ2^neg^ γδ T cells undergo rapid expansion in response to viral infection, with initial observations of this response in cytomegalovirus (CMV) infected renal allograft recipients.^152^ In solid organ transplant recipients, CMV infection is associated with higher acute rejection rates, graft damage, and opportunistic infections. There are multiple reports of a positive correlation between expansion of peripheral Vδ2^neg^ γδ T cells and resolution of CMV infection in renal allograft recipients,^153,154^ suggesting a protective antiviral role.^145,152-154^

### Natural Killer T Cells

Natural killer T (NKT) cells are an unconventional cohort of T cells displaying surface markers of the NK lineage, such as CD161 and CD94, as well as a (semi-)invariant Vαβ TCR.^155^ NKT cells recognize cognate glycolipid antigens presented via CD1d, an MHC-I-like molecule, and rapidly release proinflammatory or anti-inflammatory cytokines upon activation.^156^ Because CD1d is not polymorphic, in contrast to classical MHC molecules, alloreactive NKT cells may arguably be activated by nonspecific inflammation in a transplantation context.^157^ In contrast to conventional T cells, NKT cells are primarily localized in the liver, with smaller populations found in the lymphoid organs and peripheral circulation.^158^ Two main subsets exist, invariant/type I and type II, delineated on the basis of their TCR repertoire and lipid reactivity. The invariant NKT subset (iNKT) is the best characterized although type II NKTs appear to be the predominant subtype in humans.^159^ Influenced heavily by the nature of stimulation, NKT subsets are capable of secreting a diverse range of cytokines, including IFN-γ, TNF, IL-4, IL-10, IL-17, and IL-22.^160^

Currently, both cytolytic and protolerogenic roles have been attributed to the subset. Exemplifying this dichotomy is the observation of an exacerbating role of mouse iNKT cells in IRI, whereas type II NKTs appear to have a protective role through reduction of proinflammatory cytokines such as IFN-γ and upregulation of regulatory cytokines.^161^ iNKT cells have significant cytotoxic capacity, as evidenced by significantly decreased CD8-mediated hepatotoxicity in a murine iNKT knockout hepatic transplant model. This effect was reverted with adoptive transfer of iNKT cells.^162^ The same study noted an important role of iNKT in promoting development of a highly cytotoxic CXCR3^+^CCR4^+^CD8^+^ population, which stimulated rapid rejection of engrafted hepatocytes.^162^

In other murine transplant studies, NKT cells have displayed protolerogenic roles, prolonging sex-mismatched skin graft survival^163^ and proving essential for the establishment of corneal,^164^ cardiac,^165^ and islet graft tolerance.^166^ In a mouse bone marrow transplant model, NKT cells provoked Treg expansion and increased IL-10 secretion while decreasing IFN-γ release of donor CD4^+^ T cells,^167,168^ thereby suppressing GvHD.^169^ These studies imply far-reaching tolerogenic potential, which could serve therapeutic purposes in allotransplantation.

### Innate Lymphoid Cells

Innate lymphoid cells (ILCs) are a predominately tissue-resident population of lymphocytes that lack the diversified antigen receptors expressed on T and B cells but exert effector functions through cytokine secretion.^170^ ILCs thus represent innate counterparts of Teff cells, acting as rapid, early immune responders after activation. The major subtypes, ILC1, ILC2, and ILC3, have been described as the functional equivalent of CD4^+^ helper subsets Th1, Th2, and Th17, respectively.^171^ Consistent with this concept, transcription factor and cytokine expression of the corresponding subsets are similar.^172^ ILCs express MHC class II molecules and can process antigens, thereby regulating the activity of antigen-specific T cells.^173^

ILC1 and ILC3 cells have been attributed proinflammatory roles, whereas ILC2s primarily display immunoregulatory functions.^174,175^ Evidence within the transplant setting remains sparse; however, several studies have provided early indications of ILC involvement. Clinically, selective ILC2 subset reduction has been observed in patients with lung allograft dysfunction after reperfusion, whereas stable graft function correlated with a shift from ILC1s to ILC2s.^176^ ILC2 cells are the predominate ILC subset in the lung parenchyma, secreting type 2 cytokines (IL-4, IL-5, IL-9, and IL-13) upon airway epithelial damage, thereby contributing to type 2 inflammatory pathology.^124^ Conversely, in the setting of lung transplantation, ILC2s are activated in response to IL-33 and “danger signals” released from damaged epithelium, secreting IL-13. IL-13 has been shown to promote allograft survival because of inhibition of IL-12 and TNF-α expression by APCs.^125^ In mouse islet transplants, IL-10–producing ILC2s have also achieved prolonged graft survival, suggesting therapeutic potential.^177^

In contrast to reports of proinflammatory ILC3 function via IL-17 secretion,^178^ a protective role has recently been proposed in lung transplant recipients.^179^ ILC3-derived IL-22 appeared to promote tolerance via lymphoid tissue formation within the allograft.^179^ Similarly, long-term stability in ILC3 numbers has been associated with prolonged murine intestinal transplant survival, whereas reduced numbers were observed in unsuccessful grafts. The authors attributed this result to ILC3-secreted IL-22.^180^ These early results may indicate a potential role in allograft tolerance. A protective role of ILC3s and ILC-derived IL-22 has also been observed in allogeneic hematopoietic stem cell transplantation, with both reduction in ILC counts, especially ILC3s, and IL-22 levels associated with the onset of GvHD.^78^

In the clinical setting, ILC subsets and their precursors have demonstrated less sensitivity to immunosuppressive medication than T cells. Preserved circulating cell numbers and cytokine levels have been demonstrated in intestinal transplant patients who underwent thymoglobulin or basiliximab therapy.^181^ This phenomenon deserves further investigation and may allow prospective refinement of current treatment strategies.

## CONCLUSION

**FIGURE 2.**
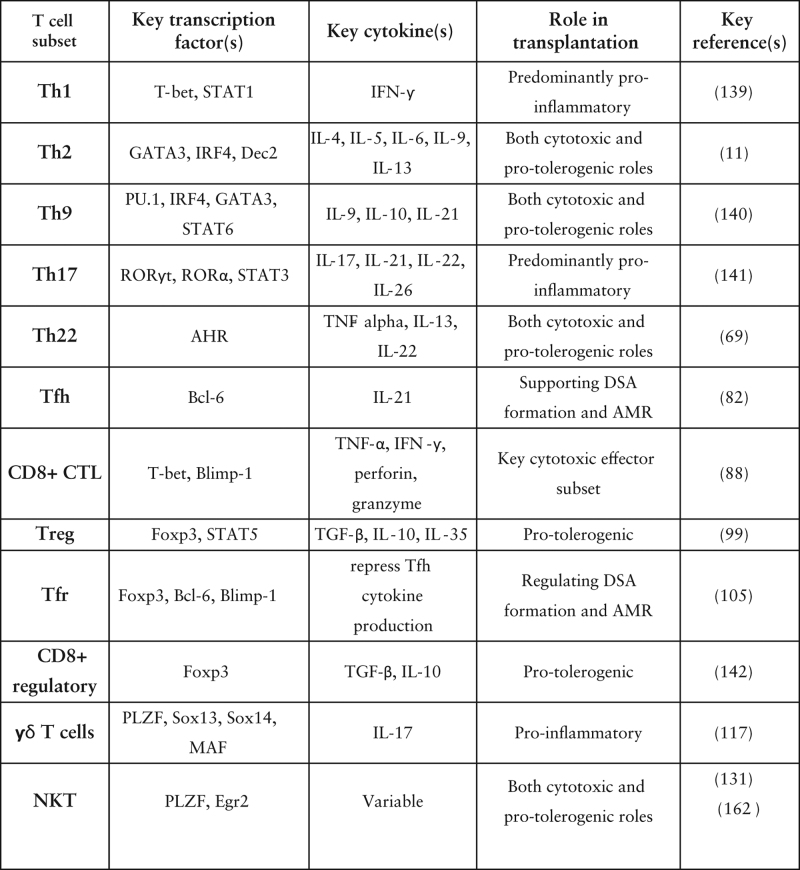
Overview of T-cell subset insights and characteristics. CTL, cytotoxic T lymphocyte; γδ, gamma-delta; IFN-γ, interferon gamma; IL, interleukin; IRF4, interferon-regulatory factor 4; NKT, natural killer T cell; ROR, RAR-related orphan nuclear receptor; Tfh, T follicular helper; Tfr, T follicular regulatory cell; TGF-β, transforming growth factor beta; Th, T helper cell; TNF-α, tumor necrosis factor alpha; Treg, regulatory T cell.

Transplant rejection is a complex response involving interactions between numerous cell types. Despite the obvious breadth of research deciphering the roles of T cell subsets in the context of rejection (Figure 2), it is clear there is still much left to understand. Thorough understanding of the relative roles of each subset, particularly their contribution to graft tolerance or rejection, is essential for the development of robust preclinical models with the potential for clinical translation. Insights into specific subset contributions may also unveil novel therapeutic targets, as highlighted throughout this review.

## ACKNOWLEDGMENTS

All figures were created with BioRender.com.
